# Understanding why oral health professionals migrate: A qualitative investigation of Iranian dentists who have moved to Canada (Oral health professionals’ migration)

**DOI:** 10.1080/16549716.2023.2190652

**Published:** 2023-04-06

**Authors:** Sara Hajian, Mohammad-Pooyan Jadidfard, Shahram Yazdani, Glen Randall, Mohammad-Hossein Khoshnevisan

**Affiliations:** a Dental Public Health Program, Department of Community Oral Health, School of Dentistry, Shaheed Beheshti University of Medical Sciences and Health Services, Tehran, IR-Iran; b Department of Community Oral Health, School of Dentistry, Shaheed Beheshti University of Medical Sciences and Health Services, Tehran, IR-Iran; c School of Health Education, Shaheed Beheshti University of Medical Sciences and Health Services, Tehran, IR-Iran; d Department of Health Policy and Management, DeGroote School of Business, McMaster University, Hamilton, Canada; e Dental Research Center, Research Institute of Dental Sciences, Preventive Dentistry Research Center, Shaheed Beheshti University of Medical Sciences and Health Services, Tehran, IR-Iran

**Keywords:** Migration, dentists, health professionals, qualitative research, low- and middle-income countries

## Abstract

**Background:**

The migration of health professionals from Low- and Middle-Income Countries (LMICs) to High-Income countries (HICs) is an ongoing phenomenon that has been accelerating with globalisation. While there has been growing research around the migration of physicians and nurses, there is less understanding of the motives surrounding the migration of dentists, and even less about their migration from specific countries.

**Objectives:**

This qualitative study explores the migration motivations of Iranian dentists who have moved to Canada.

**Methods:**

Semi-structured interviews of 18 Iranian-trained dentists in Canada were conducted to obtain information about their motives for migration. Interviews were coded and categorised into themes using qualitative thematic analysis.

**Results:**

Motivations to migrate were grouped into four analytical areas: socio-political; economic; professional; and personal. An inverse relationship was identified between the strongest motives to migrate and the topics respondents were least comfortable discussing. Socio-political-related motives were predominant with respondents focusing on their dissatisfaction with the social ethos and restrictions on personal freedom within Iran.

**Conclusion:**

Country-specific context is critical to fully understand health professional migration; in particular, the dynamics and interplay between socio-political, economic, and professional/personal factors within the home country. While Iranian dentists’ motives to migrate have some similarities to other health professionals who migrated from Iran, and dentists from other countries, differences need to be considered to fully understand migration patterns.

## Background

The migration of health professionals from Low- and Middle-Income Countries (LMICs) to High-Income Countries (HICs) is an ongoing phenomenon that has accelerated with globalisation [1]. According to the World Health Organization (WHO), there was a global shortfall of 4.3 million healthcare workers in 2006, and it is estimated that the shortage will reach 18 million by 2030 [2,3]. Iran is among the countries that face the problem of an outflow of its educated workforce, including graduates of the medical sciences [4,5]. Based on an International Monetary Fund report, Iran was ranked as the top country among 61 developing and developed nations in terms of the migration rate of highly skilled workers [5]. However, there is a lack of accurate data on the number of Iranian healthcare workers who have migrated and their country of destinations.

Health professionals migrate for a variety of reasons. Some suggest that the core motivational factors of health professional migration in LMICs are financial incentives, career development, and unqualified administrators [6]. In dentistry, understanding the reasons for migration of oral healthcare professionals remains limited compared to other healthcare professions such as medicine and nursing [7]. In Australia, 25% of dentists are internationally trained dentists who mainly come from LMICs [8]. These dentists have reported feeling let down by their home countries, particularly in terms of a lack of opportunities, and have expressed concerns about the social ethos in their home countries [8].

The majority of internationally trained dentists migrating to the United States (US) come from Asia, including the Middle East [9]. However, with the implementation of economic sanctions on Iran by the US, it has become increasingly difficult for Iranian citizens to obtain US visas, leading skilled workers, including dentists, to explore alternative destinations like Canada and Australia [10].

With 35% foreign-born doctors, Canada is among the highest in the Organisation for Economic Co-operation and Development (OECD) nations to benefit from the international migration of health professionals [11]. Moreover, Iran ranks among the top ten countries for having permanent residents in Canada during 2019, mainly through the Express Entry programme designed specifically for skilled workers [12]. However, there is currently no data available on the migration intentions of oral healthcare professionals migrating to Canada.

In Iran, dental education is separate from medical education, and admission into a dental school requires students to pass a highly competitive National University entrance examination [13]. When Iranian dentists migrate to Canada, they must establish the equivalency of their education to acquire a dental licence to practice. This can be achieved through several costly exams (direct entry) or by attending a complimentary programme at an accredited dental school (indirect entry) [14]. There is a registration deadline for each direct entry exam, and the capacity of applicants is limited [14].

Poor oral health in LMICs is mainly due to limited financial resources, inadequate coverage for preventive care, and a significant shortfall of oral health professionals [15]. As such, understanding the motives behind international dental graduates’ migration would benefit both source and destination countries by improving the efficient and equitable use of financial investments and human resources to reduce the high burden of oral diseases [16].

While there has been growing research around the migration of physicians and nurses, there is less understanding of the motives surrounding the migration of dentists, and even less about their migration from specific countries, like Iran. This qualitative study provides some preliminary information that helps to fill this research gap by exploring the specific case of Iranian-trained dentists who have moved to Canada.

## Methods

A qualitative study was conducted by exploring the life stories of Iranian-trained dentists in Canada, to identify factors that contributed to their decisions to migrate. A semi-structured questionnaire from the Australian Research Centre for Population Oral Health (ARCPOH), which was used to study the migration of dentists who moved to Australia, was adapted to be used as an interview guide for this study [8,17]. The guide includes open-ended questions and revisions took into account the Iranian context. Respondents were asked a range of questions about their motives for leaving Iran. We also conducted a literature review to identify the motivation of oral health professionals’ migration from LMICs before adapting this interview guide.

Study participants were identified through purposive sampling from a social network group of Iranian dentists in Canada that had been used to exchange experiences while attempting Canadian dental equivalency exams. One member of this group was asked to briefly introduce the study aims to the network members and request volunteers to participate in the study.

The study was approved by the Ethics Committee of the Research Institute of Dental Sciences-Shaheed Beheshti University of Medical Sciences (IR.SBMU.DRC.REC.1398.082), and McMaster University Research Ethics Board (MREB #5313). All procedures were performed in accordance with relevant approved guidelines. Before each interview, participants were provided with information about the aims of the study and were provided assurances about the anonymity of their personal information. Written or verbal informed consent was obtained from all participants. It was also verified that all participants who agreed to take part in the study met the study’s inclusion criteria; specifically, that they were living in Canada at the time of the interview for more than three years and graduated from an Iranian dental school. Participants who did not graduate from an Iranian dental school or did not begin or complete the Canadian dental equivalency programme were excluded from the study. Two participants were excluded from the study, one of them studied at a dental school outside Iran for the first two years of his education, and the other one had resided in Canada for less than three years. Interviews were conducted with individuals who volunteered to participate. No participant refused to answer any questions, and none dropped out during or after the interviews were conducted.

All interviews were undertaken in Canada, including five face-to-face interviews in a participant’s proffered café or dental practice and thirteen meetings using some virtual technology such as Zoom and WhatsApp, by the lead author between August and December 2020. All participants moved to Canada before the start of the COVID-19 pandemic. The duration of each interview was approximately one hour. After obtaining the participant’s consent, each interview was recorded digitally, and a verbatim transcript was prepared for analysis by the lead author (S.H.). Field notes highlighting important points or specific gestures, as identified by the interviewer, were written during, and immediately following, each interview. To improve accuracy, transcripts were sent to participants via email to check for any potential revision. However, no changes were identified. Data saturation was achieved after 18 interviews when no new themes emerged. Transcripts and field notes were imported to MAXQDA version 10 software. Thematic analysis was used to code the transcripts following the Braun and Clarke guideline [18,19]. Themes were derived from the data by two researchers who independently read and re-read the transcripts and notes as part of an iterative process of identifying and comparing themes between and within interviews. The themes were reviewed and categorised inductively by the research team to achieve consensus about final themes and to enhance the validity of the results.

## Results

This study aimed to explore the experiences of migrant Iranian-trained dentists who had been living in Canada for more than three years. To achieve this, the researchers conducted 18 interviews with participants aged between 30 and 56 years old, the majority of whom were female (66%). At the time of the study, 66% of participants were licenced and working as dentists in Canada, while 22% were in the process of undertaking the equivalency exams. Only two participants did not successfully pass the exams and were working in different fields. Participants lived in different parts of Canada, but most were in Toronto, the country’s most populated city. Notably, two of the participants obtained a dental speciality (post graduate degree) and three had opened their own dental clinic in Canada at the time of the interviews. Additionally, four participants obtained their speciality training from an Iranian dental school and four had owned their clinics in Iran before coming to Canada.

After thematic analysis, factors driving the migration of Iranian dentists to Canada were categorised into four main areas including ‘socio-political’, ‘economic’, ‘professional’, and ‘personal’. Within each category, several themes and sub-themes emerged and are summarised in Table 1.

**Table 1.** Factors influencing the migration of dentists from Iran.

Analytical Areas	Themes	Sub-themes
Socio-political	Restrictions on citizens’ rights	Lack of social and individual freedoms
		Religious limitations
	Disrespect for human dignity and poor social capital	Social anxiety and stress
		Gender discrimination and imposed gender roles
		Poor ethical principles based on social work core values and accountability
		Cultural norms and societal value judgments
		Interference in individuals’ privacy
	Foreign and domestic policy issues	Poor international relations and its consequences
		Difficulty in obtaining visas and travelling
		Poor physical safety in communities
		Broad political instability
		Corruption and lack of meritocracy
Economic	Devaluation of currency and inflation	Volatile remuneration (due to devaluation of currency)
		Business instability (increased cost of doing business and lack of access to some dental materials)
		Poor financial security
Professional	Unfavorable educational environment	Unfair student selection process based on biased university admission criteria
		Feeling a technology gap with developed countries
	Unclear career prospects	Shortage of postgraduate training opportunities
		Limitation for career progression and academic position
Personal	Concern for significant others	Concerns about quality of life and education for children
		Partner or family desire for migration
	Worry about own future	Lack of continuing education opportunities
		Poor income and employment prospects

## Socio-political factors

Socio-political factors were found to be the most influential factors driving migration. All participants complained about a lack of respect for their lifestyle while in Iran and pointed towards other members of their communities judging their behaviour and interfering in their personal life. One respondent noted that ‘People used to unduly intervene in your private life’ (P7). Another respondent emphasised that ‘As a dentist, I did not have serious financial or professional issues. The social and political environment in Iran was the most important factor that negatively impacted me’ (P9).

Female respondents described pressure in terms of religious oversight at the university as a major point of dissatisfaction. These pressures distracted them from their studies and had a negative impact on their overall university experience. Some of the female participants reported a perception of gender discrimination and social limitations in society. One respondent mentioned some socio-cultural taboos for women with regard to choosing their clothing and even their careers.

Most of the dissatisfaction about the work environment related back to a lack of meritocracy and favouritism regarding access to top academic and decision-making positions. A respondent suggested that ‘If the Iranian government could establish a healthy environment for supervision on enforcing the law in the society, then anyone committing a crime would know that certainly, punishment will be waiting for them … but unfortunately you can see corruption everywhere’ (P8).

Some respondents identified a work culture that did not seem to value competence in the workplace. One respondent noted that ‘Nobody did his job right! Nobody cares. Sometimes, I found a serious problem that annoyed patients. As I informed my supervisor to sort it out, I found out that he was aware of the problem for years but paid no attention to it … Everybody seems focused only on their individual self-interest’ (P2).

Meanwhile, several respondents were, in very general terms, critical of Iranian foreign policy. It was implied that some policies contributed to economic sanctions, which in turn exacerbated the poor economic situation in the country. There was also the perception that strained international relations left the country in a precarious position with citizens never knowing when the next crisis would unfold. Respondents described a general sense of never being able to let their guard down and relax. One respondent even suggested that this uncertainty included a fear of potential physical harm, noting that ‘There is always a war in the Middle East and I was concerned about another war and insecurity in Iran’ (P17).

What was most striking about the comments that fell under the socio-political theme was the initial reluctance of respondents to make comments and when they did, they tended to be vague in their descriptions of their concerns. In particular, there was an avoidance of explicit comments about the broader impact of religion or government on day-to-day life. For instance, one respondent noted that ‘As you know, the main problem is not related to dentistry. The problem is rooted in the things that we all know: the lack of political and social freedom of speech, and our problems with the world’ (P8). Once respondents began commenting on some of these issues, they indicated that just speaking about them increased their stress levels and brought them back to their time in Iran. One respondent implied that he/she was even experiencing something like post-traumatic stress just thinking about past experiences, noting that ‘Every time I think of these things, I am getting nervous as if it had just happened to me … one of my nightmares is I return to Iran and work there under those limitations … I hate it!’ (P1).

## Economic factors

Economic sanctions imposed by the American government have resulted in economic instability throughout Iran. Respondents noted that this situation had created a sense of uncertainty about the future, particularly with regard to career prospects and overall prosperity. Participants also worried about societal upheaval and increased crime due to the economic instability, which they feared could negatively impact their quality of life. A respondent lamented that ‘life becomes incredibly unpredictable in Iran’ (P15).

Of particular concern was the devaluation of Iran’s national currency and the resulting impact on businesses. One respondent emphasised the gravity of the economic instability situation noting that ‘I was always stressed with unconscious anxiety. The feeling that you don’t know what might happen tomorrow. You had to check the currency rate every day. The feeling that something unexpected is going to happen significantly affects your life’ (P13).

Another respondent stated, ‘Everything was changing in a matter of seconds; that was especially the case when I had my own office and got involved in things like buying dental equipment and materials’ (P12).

It is noteworthy to mention that most of the participants had no intention of sending money back home since the costs they endured with their move and the licencing process in Canada made them heavily indebted. However, a few participants declared their intention for supporting charity institutions back in Iran when they were financially capable.

## Professional factors

Several respondents raised concerns about educational issues. These tended to focus on problems and deficiencies in the dental student selection system and the educational environment inside dental schools. Although most participants did not have adequate knowledge of health system policies and regulations to provide detailed comments, they pointed out what they perceived as inappropriate policies that led to a recent job market saturation in urban centres and an unfair student admission system. While there is a surplus of dentists in urban areas, there remains a shortage of dentists in rural areas as dentists are reluctant to move to areas with few resources. This was pointed out by a respondent who noted that ‘The number of dentists entering the job market in Iran is still too high and competition is so fierce. I think it is justifiable that young dentists wonder about their ability to join the job market and they may rightly ask themselves why they should pursue their ambitions there [in Iran]’ (P4).

Meanwhile, participants mostly had a positive view about the quality of dental training in Iran. It was noted that, ‘When I compare Iranian education with that of other international students, I see that they [Iranians] are well-trained especially in clinical skills, for example in some fields like endodontics’ (P14). However, a technology gap between the Iranian oral healthcare system and Canada was felt by some participants. Dentists in Canada could not only be informed easily about new scientific evidence through attending continuing education courses but also could easily get access to the latest technology in their field which enables them to optimise knowledge in practice and to maintain ongoing professional development. Another respondent pointed out the variety of treatment options available to them in Canada compared to Iran, for example, the ‘Invisalign’ technology which is used in orthodontics (P13).

One other important grievance was that participants were feeling that professional and educational post-graduate training opportunities were limited and difficult to achieve. A respondent reflected on feelings of hopelessness and said ‘There is no way to progress in your own country … you cannot obtain the position for which you have studied and worked so hard. This causes you to think that it’s better not to serve your own country because no one values your abilities’ (P7).

Participants who had the experience of running their own dental office were relatively satisfied with their working environment in Iran. One respondent stated, ‘After my graduation, I opened my own office [in Iran] and I was fully satisfied with everything such as the environment and income’ (P8). Although, several respondents noted that one of the major differences between working as a dentist in Iran versus Canada was the approach to dealing with patients and the highly professional work culture in Canada.

## Personal factors

While personal factors did seem to play a role in each respondent’s reason for migrating, these reasons tended to replicate those found in the rational of individuals migrating from other professions and other countries. As a result, they may play less of a role in understanding the specific motives of Iranian-trained dentists. Respondents generally seemed most comfortable discussing their personal reasons for migrating. The concerns of participants regarding their significant others were evident, particularly for their children and spouses. In fact, the safety, education, and future prospects for their children were some of the main personal reasons that motivated their decision to migrate. As one participant shared, ‘Children can seriously impact on your decision for migration. When I was thinking about their future, I thought that my daughters will be more successful in Canada regarding their education and quality of life’ (P4). Meanwhile, another participant emphasised the importance of their spouse’s career development in their decision, stating, ‘I came to Canada with my wife because I wanted her to have better opportunities for her career. It was not just about me, but about our future together’ (P9).

Furthermore, some participants expressed worry about their own future, citing factors such as limited continuing education opportunities, poor income and employment prospects in Iran. One participant stated, ‘I was concerned about the future of my career in Iran. There were few opportunities for educational advancement, and the economic situation made it difficult to pursue my career goals’ (P3).

Despite these reasons for leaving Iran, participants also described deep and warm memories of relationships with their friends and family back home. One respondent emphasised having strong family ties in Iran and missing those relationships, stating that ‘Being in your home, passing through alleys and streets where you see your relatives, family members, and old friends who remind you of your memories, make you feel happy’ (P16).

## Discussion

This qualitative study examines the reasons several Iranian-trained dentists in Canada decided to migrate. The results show that a range of socio-political factors were the predominant reason these Iranian-trained dentists chose to migrate. They were mainly running from an unsatisfactory situation in their home country rather than the lure of Canada. Participants mainly complained about a lack of respect for their values and social rights, such as social and academic disrespect as well as interference in their private lives. Most of the problems expressed in a university context were a subset of the social dissatisfactions in the country. According to most participants, the educational environment was unfavourable and stressful concerning religious and cultural limitations. While the Iranian educational and training system, especially at high-ranking universities, was said to work well and meet high standards, socio-political dissatisfaction still acted as the main driver for migration.

Socio-political factors have been highlighted several times in studies of the Iranian ‘brain drain’. In a general study of Iranian healthcare workers, one of the most important reasons identified for migration was social factors, including restrictions on individual and social freedoms. In fact, these restrictions were so severe that many individuals reported creating dual personalities where they essentially led two lives, especially for women, so they could appear to be complying with social and cultural norms and limitations [20].

Political instability, which is mainly attributed to poor government performance related to domestic and foreign policies, was pointed out as a crucial barrier to economic development and social welfare in this study. This was a common reason for migration in other countries as well. For instance, many Lebanese physicians migrated due to political instability in Lebanon [21]. Similarly, socio-political factors, in terms of lack of civil freedom and the disappointing atmosphere after the Egyptian revolution, were among the most important driving factors for Egyptian doctors migrating to Germany [22].

Other investigators identified similar concerns expressed by internationally trained dentists in Australia, who mostly came from LMICs. They reported disappointment with being ‘let down’ in their home countries and also described their experience with a lack of professional and social ethics, such as discrimination and corruption [8]. They mostly focused on professional factors and their potential career and educational progress in Australia while in our study push factors of migration, mainly the socio-political factors, were more dominant. Health professionals migrating from Africa to the United Kingdom (UK) also pointed to their experience of ethnic tensions and discrimination in their homelands [23]. Similarly, among Iranian physicians, socio-political factors including discrimination, poor infrastructure, and lack of satisfaction with social status along with occupational factors were mentioned as the strongest influencing factors on their decision to migrate [24].

With regards to dental practices, which are mostly private entities in Iran, job satisfaction with work environment conditions also featured prominently in this study. Similar findings have also been reported as a significant driving factor in the decision for migration by other healthcare workers. One of the most crucial factors influencing the migration of physicians to Canada, including Iranian doctors, was the economic situation in their home countries [25]. Income gaps and financial incentives were also strong motivators for Caribbean physicians to migrate to the US, UK, and Canada [26]. For dentists who migrated to New Zealand, quality of life and potential income were important considerations as well [27]. However, it is important to note that while practice and financial issues were considered by Iranian dentist, they were substantially less important in the final decision for migration to Canada as compared to social factors.

Major professional concerns of study participants were the lack of postgraduate training and career progression opportunities in Iran. Similarly, career and educational opportunities were among the most important professional factors leading to migration mentioned by international doctors who migrated to the UK [28]. For Pakistani physicians, the main reasons for migration were better postgraduate education and higher salary abroad while they escaped religious intolerance, social insecurity, and terrorism in their country [29,30]. Balasubramanian et al. mentioned that ‘technology-driven but less problem-centric dental education’ in developing countries has led to migration as an escape mechanism for dentists [8]. This issue was also identified by Iranian physicians who intended to migrate, as they felt their skills were not being optimised in their home country [20].

Personal factors also had an important role in the process of migration. Family or partner decisions for migration, as well as concerns for the future of children, were among the influencing factors of migration in our study. Personal factors including the family situation and a desire for life change, personal fulfilment, and adventure were identified by healthcare workers migrating to the UK [28]. The novel experience of travel and hearing stories from family members left a deep impression on international dentists who migrated to Australia which is consistent with our findings of professionals who migrated to Canada [8].

This study has some limitations related to the nature of qualitative studies. Some examples are potential researcher bias in interpretation of the results, the possible subjectivity of interviews, and the prospect of respondents hiding information about sensitive issues. In addition, selection bias might be an issue as all participants were selected from the same social network. Thus, the results should be interpreted carefully, and the generalisability of the findings should be examined by future research.

## Conclusion

This qualitative study provides useful first-hand information about key factors motivating Iranian dentists to migrate. While Iranian dentists’ motives to migrate have some similarities to other health professionals who migrate from Iran, and to dentists from other countries, there are some differences that need to be considered to fully understand all aspects of migration patterns. In particular, Iranian dentists seem to be less concerned about the financial benefits related to migration and more focused on the need to find social and political freedom, for both them and their families, from what was described as an intolerable repression in their home country. Socio-political factors including respect for individual’s rights and dignity, personal and social freedoms, ethical values, and social security, whose control is beyond the scope of the health system alone, play more important roles in the migration of Iranian dentists. This study suggests the need for more in-depth and nuanced studies that evaluate the migration of health professionals in order to better understand and help predict evolving migration patterns.

## Paper context

Large numbers of health professionals have been migrating from low-income to high-income countries. While there is some research around the rationale and subsequent experiences of physicians, little is known about why dentists migrate, and almost nothing about why dentists from Iran migrate. This paper helps fill that gap by providing insights into why Iranian dentists migrate to Canada and identifies what makes them different from other health professionals and, in particular, dentists from other countries.
